# Fidelity-agnostic synthetic data generation improves utility while retaining privacy

**DOI:** 10.1016/j.patter.2025.101287

**Published:** 2025-06-05

**Authors:** Jim Achterberg, Marcel Haas, Bram van Dijk, Marco Spruit

**Affiliations:** 1 Public Health and Primary Care (Health Campus The Hague), Leiden University Medical Center, Leiden, South-Holland, the Netherlands; 2 Leiden Institute of Advanced Computer Science, Leiden University, Leiden, South-Holland, the Netherlands

**Keywords:** privacy, machine learning, responsible AI, generative AI, data synthesis, data anonymization, representation learning, supervised learning

## Abstract

Synthetic data are a popular method to publish useful datasets in a privacy-aware manner, making them useful across a range of scientific domains involving human subjects. They are typically generated by sampling from algorithms that mimic the probability distribution of real datasets, thereby maximizing statistical similarity to real data. However, we argue and demonstrate that synthetic data need to be similar only in ways *relevant* to their intended use and may neglect any *irrelevant* information, which in turn may improve privacy protection. As such, we propose a data synthesis method entitled fidelity-agnostic synthetic data. The method first extracts features relevant to the dataset’s intended use using a neural net and then generates synthetic versions of the extracted features, after which they are decoded to mimic the real dataset. We show that our synthetic data improve performance in prediction tasks while retaining privacy protection compared to other state-of-the-art methods.

## Highlights

• Synthetic data can be useful for prediction without closely resembling real data
• Less resemblance between synthetic and real data improves privacy
• Our synthetic data are more useful in prediction while maintaining privacy

## The bigger picture

Access to data is essential in driving scientific progress. Unfortunately, privacy concerns often prevent the sharing of real-world datasets, limiting researchers’ access to data. Synthetic data—artificially generated data that mimic real datasets—offer a potential solution. Traditionally, synthetic data are created to closely resemble real datasets, but this can risk exposing private information. Our research introduces a new approach that prioritizes usefulness for specific tasks, such as predictive modeling, without directly imitating the original data. Using four real-world datasets where privacy concerns might apply, we demonstrate that our method produces synthetic data that are more effective for prediction while maintaining strong privacy protection. This makes our method suitable for mitigating privacy concerns in a wide variety of applications that rely on prediction, e.g., in digital health, epidemiology, and biomedical research.

## Teaser

Data accessibility is often limited due to privacy concerns, hindering scientific progress. Algorithmically generated synthetic data are a potential solution. Typically, they are generated to closely resemble a real dataset, such that they are similarly useful as the real data. However, close resemblance can also risk exposing sensitive information. This article introduces a new method for synthesizing data that optimizes directly for usefulness in specific tasks instead of overall resemblance to real data, thereby enhancing usefulness while maintaining privacy protection.

## Introduction

With a rising number of data-driven applications built on personal data, synthetic data (SD) boast considerable potential to decrease associated privacy risks. This makes SD a promising technology for a wide variety of disciplines that rely on sensitive human data, including medical, social, educational, and financial sciences.^1^ The goal of SD is to generate a dataset that can be used instead of real data (RD) when the latter cannot be used due to ethical, legal, privacy, or other concerns. Since SD are used in real tasks, and often to mitigate privacy concerns, they should be generated to be similarly useful as RD while leaking as little real, sensitive information as possible.^2^^,^^3^ If successful, SD can serve a variety of privacy-preserving tasks, e.g., federated learning through sharing data instead of model parameters^4^ and open sourcing research data^1^; we mainly focus on the latter.

There is often a trade-off between SD usefulness and their privacy-preserving capabilities, also referred to as the utility-privacy trade-off.^5^^,^^6^^,^^7^^,^^8^ For SD to be useful, they are typically generated to be statistically similar to RD, i.e., to have *high fidelity*. High-fidelity SD may degrade privacy preservation, however, since they may increase the risk of reidentification. Statistical patterns in the SD may be used to infer real sensitive information, or individual SD points may be matched to RD directly.^9^

Training algorithms that generate SD typically involves minimizing the statistical divergence between generated samples and RD. In other words, they generate *high-fidelity* SD. We argue that this is not the best strategy in some cases, especially when the specific task the SD will perform, e.g., prediction, is known beforehand. Generating SD to have *high utility* in said task, without optimizing for fidelity, may improve privacy preservation.

Retaining high utility without optimizing for fidelity is possible, since fidelity metrics alone are not a reliable predictor of SD utility in specific tasks. For example, when metrics that do not account for low SD variety indicate high fidelity, utility may still be poor.^10^ But, equally important, poor fidelity does not always imply poor utility. Rarely are all statistical patterns present in RD relevant to a specific task, and discrepancies in irrelevant patterns that reduce fidelity do not necessarily affect utility. In fact, neglecting irrelevant patterns may lead to more dissimilarity between SD and RD, which in turn may lead to a reduced risk of reidentification. However, this mostly depends on the specific dataset and which features are deemed sensitive or identifiable and how influential those features are in the task at hand.

Several existing approaches focus on generating SD tailored to specific tasks, prioritizing high utility. Zhao et al.^11^ guide the representation space of generative models to increase SD utility, but still mainly optimize for high fidelity. Liu et al.^12^ and Räisä et al.^13^ generate SD for private query release in discrete tabular datasets. More similar to our work, Chen et al.^14^ generate SD for supervised prediction by optimizing SD to yield similar weights when training downstream neural net classifiers. Since SD are generated by backpropagating gradients through a synthetic set, which is a continuous process, their method is mainly tailored toward numerical rather than discrete or mixed-type datasets.

**Figure 1:**
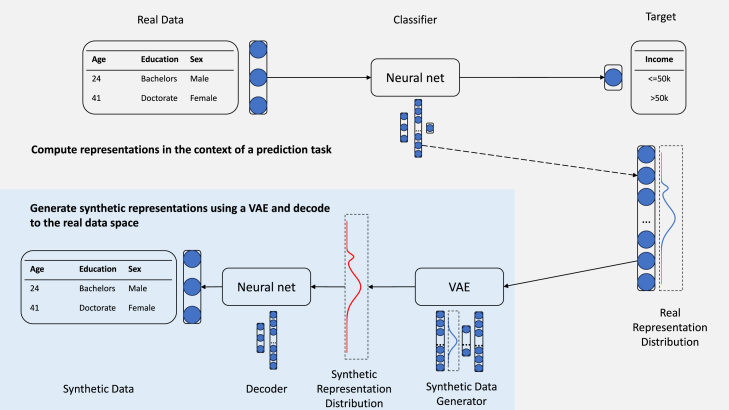
Schematic overview of the FASD generation process

To generate SD for high utility directly, we draw inspiration from the field of representation learning.^15^ We compute representations of the RD by passing them through the encoder portion of a neural net, i.e., all layers except the prediction head, which we train to predict a target feature. This process extracts valuable information while neglecting irrelevant information (to the prediction task) in the data. Then, we use these representations as input to an SD-generating algorithm, i.e., a variational auto-encoder (VAE),^16^ to generate synthetic representations. Finally, we train a neural decoder to decode synthetic representations back to the original data space, to obtain a set of SD. The final set of SD thus has the same structure as the RD and is generated to be similar to RD *only* in those aspects that contribute to its usefulness in a prediction task. This process constitutes our method for SD generation and we will refer to it as fidelity-agnostic synthetic data (FASD) in the rest of this paper. Figure 1 provides a schematic overview of FASD (visualization inspired by that of Zhang et al.^17^).

We compare FASD with SD generated through standard approaches for high fidelity, across four tabular datasets originating from various domains where privacy concerns may be relevant. Here, we evaluate a wide range of metrics to investigate the quality and privacy-preserving properties of the generated datasets. Although we consider only tabular data, our approach is extendable to other data modalities as well.

## Results

### Benchmarking

We benchmark FASD against other SD generators to assess the quality of generated SD. Typically, evaluation of SD quality comprises three main aspects: fidelity, utility, and privacy risk.^18^^,^^19^ We provide a brief summary of all evaluation metrics incorporated into the benchmark in Table 1.

**Table 1:** Summary of evaluation metrics

	Metric	Summary	Direction
Fidelity	JS	measures similarity between distributions	↓
	α-precision^7^	measures degree of coverage of the SD distribution by RD	↓
	β-recall^7^	measures degree of coverage of the RD distribution by SD	↓
	distinguishability	measures the AUROC of a classifier (XGBoost) that aims to distinguish SD from RD	↓
Utility	TSTR	measures the AUROC (one-versus-rest microaveraged in multiclass scenario) of prediction models (linear regression, XGBoost, and neural net) trained on SD and tested on RD	↑
	feature importance	measures correlation between feature importances from an XGBoost model trained on SD and RD	↑
Privacy	k-map^20^	measures minimum amount of similar samples in SD compared to RD with respect to sensitive features	↑
	δ-presence^21^	measures maximum ratio of similar samples, with respect to sensitive features, in SD compared to RD	↑
	authenticity^7^	measures the frequency of RD that is closer to other RD than to SD, e.g., by Euclidean distance	↑
	identifiability^22^	measures the frequency of RD that is closer to SD than to other RD (opposite of authenticity), where distances are weighted such that rare values are seen as more identifiable	↓
	attribute inference	AUROC (discrete) or $R^{2}$ (continuous) score of a prediction model (XGBoost) trained to infer sensitive from non-sensitive features in SD, tested on RD	↓
	membership inference	AUROC of an inference model (DOMIAS^23^) aiming to infer which RD points were used to train the SD generator	↓

Direction indicates whether higher (↑) or lower (↓) values are desirable.

Fidelity (also called “global” or “broad” utility^24^) metrics assess SD realism, either through human evaluation or, more commonly, through statistical analysis. Due to their similarity to RD, high-fidelity SD can typically be used in a wide variety of tasks and achieve results comparable to those of RD. We use Jensen-Shannon (JS) distance, α-precision β-recall,^7^ and classifier distinguishability^19^ to measure SD fidelity.

Utility (also called “narrow” or “analysis-specific” utility^24^) metrics assess whether SD can be used in a *specific task* or *set of tasks* and therein achieve results comparable to those of RD. Arguably, both fidelity and utility metrics indicate SD usefulness, where fidelity indicates general usefulness for a set of *unknown* tasks and utility indicates specific usefulness for a set of *known* tasks. Supervised learning tasks are most commonly considered to assess SD utility,^11^^,^^19^^,^^22^^,^^25^^,^^26^^,^^27^^,^^28^^,^^29^^,^^30^^,^^31^^,^^32^ which we follow. We use the train synthetic test real (TSTR)^33^ framework to assess SD utility in a prediction task, which trains a prediction model on SD and compares with one trained on RD and evaluates both on an independent test set of RD. Additionally, we provide the similarity in feature importances from SD and RD. This provides insight into whether SD and RD achieve predictions from input features in similar fashions.

Privacy metrics indicate the risk of identifying real sensitive information from SD either by disclosing additional information (attribute disclosure) or disclosing which RD were used to train the SD generator (membership disclosure).^2^ Attribute disclosure may occur when some features in SD closely match features in RD that are openly available, after which additional information can be determined from SD. Otherwise, attribute disclosure can also occur when attackers train prediction models to exploit statistical patterns present in SD to predict sensitive information in RD.^19^ Membership disclosure can occur when the SD generator overfits to RD points, allowing attackers to detect which points were used during training. We use k-map,^20^ δ-presence,^21^ authenticity,^7^ identifiability,^22^ attribute inference risk,^19^ and membership inference risk^23^ as privacy metrics. For attribute inference risk, we report the average rank of inference accuracy over all features, since average accuracy scores tend to be dominated by individual features in the case of low $R^{2}$ scores.

Similar to Yan et al.,^8^ we rank the evaluation metric scores of each of the five SD generators from 1 (best) to 5 (worst) for each dataset. By reporting ranks of metrics instead of the metrics themselves, we can display the performance of SD generators across different metrics in a single figure, thereby providing an indication of overall performance. However, unlike Yan et al.,^8^ we statistically test differences in overall performance and do not collapse the performance in terms of fidelity, utility, and privacy into a single score. This way, we can indicate how FASD maintains high utility and privacy protection while potentially degrading fidelity.

We compare SD quality from FASD with the SD generators TVAE,^31^ CTGAN,^31^ AdsGAN,^22^ and DP-GAN.^34^ TVAE and CTGAN are popular “general-purpose” SD generators, i.e., they generate SD with the highest possible fidelity. Since our goal is to retain privacy while achieving high utility, we also include AdsGAN and DP-GAN, which have this same goal of privacy preservation at heart. Here, AdsGAN focuses on practical rather than formal privacy guarantees, whereas DP-GAN provides formal guarantees through differential privacy.^35^ We perform all SD generation and evaluation using the SynthCity library,^36^ which we extended with the FASD methodology and several other alterations to fit the methods and metrics described in this article. We perform the benchmarking across 10 random folds of real training data, with an 80-20 train-validation split stratified on the target feature.

### Datasets

We consider four different datasets for benchmarking. Inclusion criteria for the datasets were that they:

(1) are publicly available,
(2) are in a tabular format,
(3) contain both numerical and discrete data types,
(4) contain information on human subjects that may be deemed sensitive, and
(5) originate from a variety of scientific disciplines.

As such, we consider four datasets from the University of California, Irvine, Machine Learning (UCI ML) repository,^37^ abbreviated as adult, credit, student, and heart. Table 2 contains general information on all four datasets. For the sake of preventing arbitrary choices, we assume that any feature may be deemed sensitive or identifiable, such that they are all included in the privacy assessments.

**Table 2:** Characteristics of benchmarking datasets

	Adult	Credit	Student	Heart
*n*	48,842	30,000	4,424	303

**Number of features**				

Numerical	4	14	19	5
Discrete	7	9	17	8
Target feature	income >50k	default	graduate	heart disease
Label proportions	76% no, 24% yes	78% no, 22% yes	32% no, 18% delay, 50% yes	54% no, 46% yes

y% x indicates a frequency y of corresponding label x.

#### Adult

The adult census dataset was extracted from the 1994 Census Bureau database and contains demographic, economic, and population data from the US Census Bureau. The prediction task is to classify whether a person earns over $50,000 annually. The dataset has been used extensively to benchmark methods relating to SD generation and evaluation.^12^^,^^25^^,^^27^^,^^38^^,^^39^^,^^40^^,^^41^

#### Credit

The default of credit card clients dataset, introduced in Yeh and Lien,^42^ contains credit card payment data linked to demographic information from a Taiwanese bank during October 2005. The prediction task is whether a credit card client will default on their payment in the next period. This dataset has also been used previously in SD literature.^26^^,^^43^

#### Student

The students dropout and academic success dataset, introduced in Martins et al.,^44^ contains data on undergraduate students of the Polytechnic Institute of Portalegre, Portugal, between 2008 and 2019. It contains extensive demographic and personal information, previous academic performances, and features indicating the state of the economy. The prediction task is whether a student obtained a degree in due time, with a delay up to 3 years, or not at all.

#### Heart

The heart disease dataset, introduced in Detrano et al.,^45^ contains patients referred for coronary angiography at the Cleveland Clinic between May 1981 and September 1984. The dataset contains vital sign measurements, lab results, and demographic information. The prediction task is whether any presence of heart disease is detected. The heart disease dataset has been used previously in SD generation.^28^^,^^29^^,^^46^

### Utility-privacy trade-off

**Figure 2:**
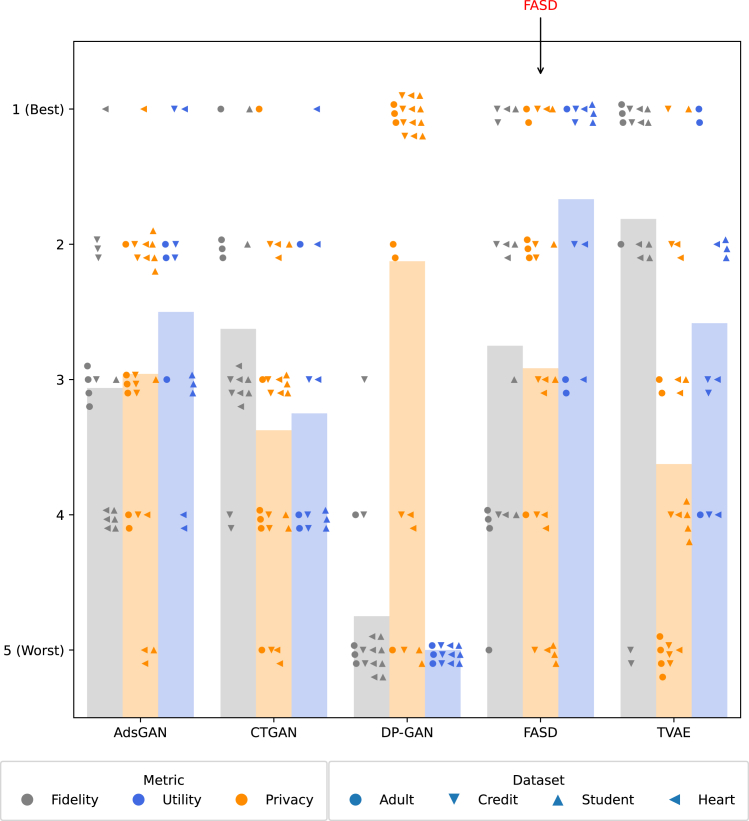
Ranks of evaluation metric scores on fidelity, utility, and privacy of SD Each point corresponds to the ranking of a metric for a specific dataset for the respective SD generator versus the other SD generators. Vertical jitter is added to improve visibility of individual points. Bars indicate average ranking per SD generator.

Figure 2 provides a general overview on how FASD ranks against other SD generators in terms of fidelity, utility, and privacy across all datasets. Note that utility includes only TSTR and not feature importances, since we mainly care about prediction performance in this article. SD generators are ranked from 1 (best) to 5 (worst) for each evaluation metric in each dataset; tied ranks are averaged. Although fidelity metrics are not directly of interest, since we care only about utility in a certain task, we include them to further investigate the properties of SD generators. Tables 3, 4, and 5 provide a more in-depth view on the benchmarking results.

**Table 3:** Fidelity of synthetic data

Metric	SD generator	Adult	Credit	Student	Heart
JS ↓	AdsGAN	$0.007_{\pm 0.002}$	$0.007_{\pm 0.002}$	$0.010_{\pm 0.001}$	$0.020_{\pm 0.002}$
	CTGAN	$0.005_{\pm 0.001}$	$0.008_{\pm 0.002}$	$0.009_{\pm 0.001}$	$0.021_{\pm 0.003}$
	DP-GAN	$0.091_{\pm 0.012}$	$0.078_{\pm 0.007}$	$0.079_{\pm 0.007}$	$0.085_{\pm 0.005}$
	FASD	$0.008_{\pm 0.001}$	$0.005_{\pm 0.000}$	$0.010_{\pm 0.000}$	$0.022_{\pm 0.002}$
	TVAE	$0.005_{\pm 0.001}$	$0.003_{\pm 0.000}$	$0.008_{\pm 0.001}$	$0.021_{\pm 0.002}$
Distinguishability ↓	AdsGAN	$0.926_{\pm 0.042}$	$0.795_{\pm 0.050}$	$0.971_{\pm 0.005}$	$0.710_{\pm 0.059}$
	CTGAN	$0.901_{\pm 0.058}$	$0.900_{\pm 0.035}$	$0.951_{\pm 0.037}$	$0.705_{\pm 0.094}$
	DP-GAN	$1.000_{\pm 0.000}$	$1.000_{\pm 0.000}$	$0.999_{\pm 0.001}$	$0.966_{\pm 0.049}$
	FASD	$1.000_{\pm 0.000}$	$0.908_{\pm 0.022}$	$0.983_{\pm 0.007}$	$0.702_{\pm 0.041}$
	TVAE	$0.879_{\pm 0.028}$	$0.652_{\pm 0.014}$	$0.961_{\pm 0.015}$	$0.580_{\pm 0.056}$
α-precision ↑	AdsGAN	$0.771_{\pm 0.172}$	$0.684_{\pm 0.351}$	$0.924_{\pm 0.031}$	$0.609_{\pm 0.133}$
	CTGAN	$0.820_{\pm 0.146}$	$0.414_{\pm 0.362}$	$0.927_{\pm 0.034}$	$0.705_{\pm 0.130}$
	DP-GAN	$0.134_{\pm 0.097}$	$0.287_{\pm 0.230}$	$0.600_{\pm 0.219}$	$0.447_{\pm 0.268}$
	FASD	$0.286_{\pm 0.042}$	$0.861_{\pm 0.024}$	$0.966_{\pm 0.016}$	$0.788_{\pm 0.073}$
	TVAE	$0.941_{\pm 0.025}$	$0.181_{\pm 0.268}$	$0.974_{\pm 0.007}$	$0.830_{\pm 0.115}$
β-recall ↑	AdsGAN	$0.294_{\pm 0.094}$	$0.342_{\pm 0.159}$	$0.391_{\pm 0.025}$	$0.423_{\pm 0.100}$
	CTGAN	$0.333_{\pm 0.080}$	$0.241_{\pm 0.207}$	$0.398_{\pm 0.027}$	$0.440_{\pm 0.099}$
	DP-GAN	$0.028_{\pm 0.048}$	$0.242_{\pm 0.195}$	$0.019_{\pm 0.012}$	$0.094_{\pm 0.100}$
	FASD	$0.016_{\pm 0.006}$	$0.514_{\pm 0.007}$	$0.483_{\pm 0.018}$	$0.512_{\pm 0.106}$
	TVAE	$0.364_{\pm 0.025}$	$0.106_{\pm 0.127}$	$0.474_{\pm 0.013}$	$0.476_{\pm 0.056}$

x ± y indicates a mean result of x and standard deviation of y for results across 10 folds of training data. Arrows indicate whether higher (↑) or lower (↓) values are desirable, and best results are in bold.

**Table 4:** Utility of synthetic data

Metric	SD generator	Adult	Credit	Student	Heart
Linear regression ↑	real data	$0.904_{\pm 0.003}$	$0.766_{\pm 0.008}$	$0.910_{\pm 0.012}$	$0.909_{\pm 0.020}$
	AdsGAN	$0.886_{\pm 0.003}$	$0.750_{\pm 0.010}$	$0.861_{\pm 0.010}$	$0.740_{\pm 0.106}$
	CTGAN	$0.884_{\pm 0.003}$	$0.741_{\pm 0.011}$	$0.860_{\pm 0.011}$	$0.812_{\pm 0.070}$
	DP-GAN	$0.533_{\pm 0.121}$	$0.555_{\pm 0.065}$	$0.499_{\pm 0.081}$	$0.574_{\pm 0.157}$
	FASD	$0.885_{\pm 0.004}$	$0.749_{\pm 0.014}$	$0.919_{\pm 0.032}$	$0.774_{\pm 0.061}$
	TVAE	$0.897_{\pm 0.003}$	$0.746_{\pm 0.006}$	$0.883_{\pm 0.011}$	$0.786_{\pm 0.115}$
Neural net ↑	real data	$0.908_{\pm 0.002}$	$0.768_{\pm 0.007}$	$0.902_{\pm 0.011}$	$0.902_{\pm 0.039}$
	AdsGAN	$0.826_{\pm 0.098}$	$0.648_{\pm 0.078}$	$0.841_{\pm 0.010}$	$0.692_{\pm 0.083}$
	CTGAN	$0.860_{\pm 0.005}$	$0.606_{\pm 0.063}$	$0.833_{\pm 0.010}$	$0.646_{\pm 0.128}$
	DP-GAN	$0.483_{\pm 0.030}$	$0.506_{\pm 0.018}$	$0.516_{\pm 0.065}$	$0.532_{\pm 0.147}$
	FASD	$0.867_{\pm 0.004}$	$0.715_{\pm 0.039}$	$0.877_{\pm 0.034}$	$0.688_{\pm 0.072}$
	TVAE	$0.739_{\pm 0.116}$	$0.576_{\pm 0.013}$	$0.852_{\pm 0.011}$	$0.643_{\pm 0.131}$
XGBoost ↑	real data	$0.927_{\pm 0.003}$	$0.780_{\pm 0.007}$	$0.921_{\pm 0.007}$	$0.875_{\pm 0.021}$
	AdsGAN	$0.866_{\pm 0.010}$	$0.699_{\pm 0.029}$	$0.851_{\pm 0.014}$	$0.671_{\pm 0.123}$
	CTGAN	$0.858_{\pm 0.016}$	$0.690_{\pm 0.022}$	$0.851_{\pm 0.021}$	$0.748_{\pm 0.087}$
	DP-GAN	$0.541_{\pm 0.087}$	$0.509_{\pm 0.070}$	$0.528_{\pm 0.112}$	$0.614_{\pm 0.098}$
	FASD	$0.865_{\pm 0.014}$	$0.708_{\pm 0.024}$	$0.909_{\pm 0.027}$	$0.773_{\pm 0.068}$
	TVAE	$0.876_{\pm 0.007}$	$0.696_{\pm 0.022}$	$0.882_{\pm 0.012}$	$0.712_{\pm 0.089}$
Feature importance^a^↑ (from XGBoost)	AdsGAN	$0.426_{\pm 0.052}$	$0.243_{\pm 0.048}$	$0.343_{\pm 0.092}$	$0.141_{\pm 0.143}$
	CTGAN	$0.378_{\pm 0.090}$	$0.192_{\pm 0.059}$	$0.336_{\pm 0.077}$	$-0.038_{\pm 0.221}$
	DP-GAN	$-0.001_{\pm 0.169}$	$-0.052_{\pm 0.131}$	$-0.212_{\pm 0.169}$	$0.026_{\pm 0.356}$
	FASD	$0.374_{\pm 0.075}$	$0.158_{\pm 0.086}$	$0.518_{\pm 0.035}$	$-0.031_{\pm 0.233}$
	TVAE	$0.484_{\pm 0.065}$	$0.272_{\pm 0.056}$	$0.398_{\pm 0.096}$	$0.051_{\pm 0.202}$

x ± y indicates a mean result of x and standard deviation of y for results across 10 folds of training data. Arrows indicate whether higher (↑) or lower (↓) values are desirable, and best results are in bold.

a Excluded from Figure 2.

**Table 5:** Privacy risk of synthetic data

	SD generator	Adult	Credit	Student	Heart
*k*-map ↑	AdsGAN	${2,368.200}_{\pm 381.855}$	${1,244.500}_{\pm 550.859}$	$172.000_{\pm 20.885}$	$25.900_{\pm 3.506}$
	CTGAN	${2,408.400}_{\pm 229.709}$	${1,108.100}_{\pm 391.740}$	$149.100_{\pm 21.714}$	$24.000_{\pm 4.290}$
	DP-GAN	${1,131.100}_{\pm 1,365.333}$	$803.000_{\pm 928.134}$	$65.300_{\pm 31.135}$	$24.300_{\pm 4.562}$
	FASD	${1,931.700}_{\pm 267.475}$	$709.400_{\pm 90.123}$	$195.800_{\pm 138.104}$	$25.100_{\pm 3.330}$
	TVAE	${2,342.300}_{\pm 82.445}$	${1,306.300}_{\pm 161.931}$	$155.300_{\pm 20.445}$	$25.200_{\pm 3.124}$
δ-presence ↑	AdsGAN	$1.110_{\pm 0.199}$	$1.509_{\pm 0.668}$	$1.100_{\pm 0.055}$	$1.157_{\pm 0.123}$
	CTGAN	$1.047_{\pm 0.058}$	$1.435_{\pm 0.466}$	$1.137_{\pm 0.063}$	$1.248_{\pm 0.194}$
	DP-GAN	$86.391_{\pm 180.420}$	$159.174_{\pm 267.080}$	$5.471_{\pm 2.883}$	$1.311_{\pm 0.322}$
	FASD	$1.236_{\pm 0.188}$	$1.905_{\pm 0.268}$	$1.745_{\pm 1.206}$	$1.221_{\pm 0.136}$
	TVAE	$1.021_{\pm 0.014}$	$1.087_{\pm 0.081}$	$1.111_{\pm 0.073}$	$1.189_{\pm 0.104}$
Authenticity ↑	AdsGAN	$0.612_{\pm 0.071}$	$0.404_{\pm 0.251}$	$0.563_{\pm 0.026}$	$0.513_{\pm 0.059}$
	CTGAN	$0.583_{\pm 0.053}$	$0.207_{\pm 0.246}$	$0.544_{\pm 0.021}$	$0.530_{\pm 0.032}$
	DP-GAN	$0.959_{\pm 0.062}$	$0.737_{\pm 0.297}$	$0.971_{\pm 0.021}$	$0.869_{\pm 0.103}$
	FASD	$0.976_{\pm 0.023}$	$0.566_{\pm 0.012}$	$0.503_{\pm 0.017}$	$0.520_{\pm 0.045}$
	TVAE	$0.568_{\pm 0.019}$	$0.059_{\pm 0.155}$	$0.508_{\pm 0.013}$	$0.521_{\pm 0.068}$
Identifiability ↓	AdsGAN	$0.312_{\pm 0.102}$	$0.226_{\pm 0.209}$	$0.388_{\pm 0.022}$	$0.420_{\pm 0.115}$
	CTGAN	$0.344_{\pm 0.083}$	$0.176_{\pm 0.193}$	$0.408_{\pm 0.023}$	$0.431_{\pm 0.090}$
	DP-GAN	$0.016_{\pm 0.015}$	$0.043_{\pm 0.054}$	$0.020_{\pm 0.011}$	$0.077_{\pm 0.059}$
	FASD	$0.021_{\pm 0.006}$	$0.318_{\pm 0.125}$	$0.484_{\pm 0.020}$	$0.538_{\pm 0.091}$
	TVAE	$0.375_{\pm 0.036}$	$0.330_{\pm 0.214}$	$0.480_{\pm 0.023}$	$0.548_{\pm 0.064}$
Attribute inference (rank)^a^↓	AdsGAN	$4.000_{\pm 0.877}$	$3.692_{\pm 0.970}$	$3.436_{\pm 0.940}$	$3.812_{\pm 0.834}$
	CTGAN	$3.643_{\pm 0.497}$	$3.769_{\pm 1.107}$	$3.282_{\pm 1.099}$	$3.062_{\pm 0.998}$
	DP-GAN	$1.286_{\pm 0.469}$	$1.000_{\pm 0.000}$	$1.026_{\pm 0.160}$	$1.000_{\pm 0.000}$
	FASD	$1.714_{\pm 0.469}$	$3.577_{\pm 1.270}$	$3.923_{\pm 1.222}$	$4.125_{\pm 1.258}$
	TVAE	$4.357_{\pm 0.929}$	$2.962_{\pm 0.999}$	$3.333_{\pm 1.177}$	$3.000_{\pm 1.033}$
Membership inference ↓	AdsGAN	$0.505_{\pm 0.001}$	$0.501_{\pm 0.002}$	$0.503_{\pm 0.006}$	$0.542_{\pm 0.032}$
	CTGAN	$0.505_{\pm 0.003}$	$0.503_{\pm 0.003}$	$0.505_{\pm 0.006}$	$0.564_{\pm 0.029}$
	DP-GAN	$0.501_{\pm 0.003}$	$0.511_{\pm 0.003}$	$0.514_{\pm 0.011}$	$0.560_{\pm 0.059}$
	FASD	$0.501_{\pm 0.003}$	$0.501_{\pm 0.003}$	$0.504_{\pm 0.008}$	$0.540_{\pm 0.030}$
	TVAE	$0.504_{\pm 0.001}$	$0.503_{\pm 0.003}$	$0.498_{\pm 0.006}$	$0.547_{\pm 0.018}$

x ± y indicates a mean result of x and standard deviation of y for results across 10 folds of training data. Arrows indicate whether higher (↑) or lower (↓) values are desirable, and best results are in bold.

a Average rank of inference accuracy over all features.

FASD outranks other SD generators in terms of utility and all except DP-GAN in terms of privacy, albeit slightly, with DP-GAN achieving poor utility. This suggests that FASD is able to improve upon the utility-privacy trade-off when compared to the other SD generators, by increasing utility while retaining similar privacy levels. Simultaneously, FASD performs only moderately in terms of fidelity. This indicates that the method performs as hypothesized: we can achieve high utility and adequate privacy protection by retaining only those statistical patterns that are relevant to the specific task at hand.

Ranking metric scores provides a straightforward way to test the significance of results through Mann-Whitney U tests. In terms of utility, FASD significantly outranks DP-GAN $\left(p_{adj}=0.000\right)$, CTGAN $\left(p_{adj}=0.003\right)$, TVAE $\left(p_{adj}=0.040\right)$, and AdsGAN $\left(p_{adj}=0.040\right)$ on a 5% level. In terms of privacy there is no significant difference in two-tailed tests on a 5% level between FASD and DP-GAN $\left(p_{adj}=0.144\right)$, TVAE $\left(p_{adj}=0.333\right)$, CTGAN $\left(p_{adj}=0.492\right)$, and AdsGAN $\left(p_{adj}=0.775\right)$. Here, $p_{adj}$ indicates adjusted p values through Holm-Bonferroni correction.^47^

#### Results for utility

Multiple factors might a play a role in the high utility performance of FASD. First, by extracting only features relevant to the prediction task, we simplify the learning space of the SD generator from a relatively complex heterogeneous feature space to a more densely sampled continuous space. In general, generative algorithms are better suited to learning these types of feature spaces.^48^ Second, by ensuring the SD generator is required to learn only relevant patterns and neglect irrelevant ones, the potential of overfitting to irrelevant patterns is reduced, promoting SD generation that is more robust in terms of its utility.

More specifically, although overall fidelity of FASD is low, we expect features that are major joint contributors to representations extracted by the FASD encoder to have (relatively) high fidelity. These features preserve associations with other features through these representations and propagate them to SD via the decoder. This insight has major implications for the feature importance and attribute inference metric. In terms of feature importances, the importance ranking of these features will be similar to that in RD, as their joint distribution can be accurately decoded. For other, less important features, decoding and thus feature importances are more random. This can be seen from Table 4, as even when FASD performs best in terms of predictive performance, feature importances are not necessarily preserved the best.

**Figure 3:**
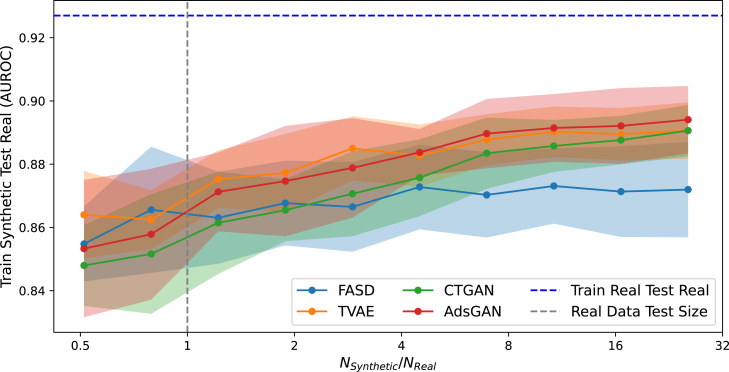
Train synthetic test real performance (from XGBoost) against relative synthetic data sample size for the adult census dataset Average performance across 10 folds, error margins indicate standard deviation.

Table 4 shows SD utility when sampling SD of the same size as RD. However, we can synthesize larger datasets as well, which can potentially increase utility in predictive tasks. Sampling additional data can improve diversity and increase the capacity of predictive models to accurately learn conditional label distributions.^49^ Diversity in conditional label distributions can be especially well assessed through the TSTR approach; the inverse procedure, training on RD and testing on SD, indicates only whether *realistic* labels are generated, not whether they are *diverse*.^33^ Figure 3 shows TSTR performance for an increasing SD size (relative to RD test size). We omit DP-GAN from this analysis due to its low utility to enhance readability of the plot. Performance increases more for other SD generators than for FASD when SD size increases. With its task-specific focus, FASD is less capable of increasing data diversity than other SD generators that aim to mimic the full RD distribution; therefore the gains of increasing sample size are lowered as well. However, as Table 4 shows, FASD outranks other SD generators in terms of utility when SD and RD are of similar size; this is the commonly considered scenario in SD literature. Increasing SD size can enhance utility, but it can increase privacy leakage as well by amplifying risk sources such as overfitting.^50^

#### Results for privacy

**Figure 4:**
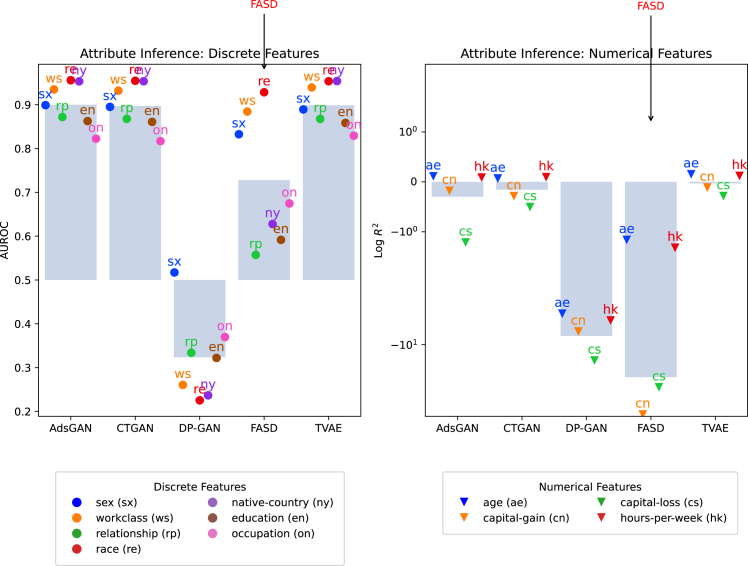
Attribute inference accuracy (from XGBoost) for the adult census dataset

In terms of privacy, FASD especially provides potential gains for those features that do not contribute (jointly) to the representations and are therefore not preserved well in terms of fidelity. We show this more clearly by investigating attribute inference risk for individual features for the adult dataset in Figure 4. Here, indeed, we see that FASD is more protective for certain attributes than others. Contrarily, methods that provide *general* protectiveness, like DP-GAN, do so for all features, but potentially at the cost of overall low fidelity.

Interestingly, Figure 4 also indicates how FASD would fare in terms of utility for predicting features it was not optimized for. Namely, utility is especially poor for those features that are neglected by the FASD encoder in the prediction task.

For other privacy metrics, FASD achieves privacy gain, since some features have low fidelity, causing less similarity on a sample level. This is relevant for metrics that indicate sample-level similarity between SD and RD, e.g., authenticity and identifiability, and metrics that indicate overfitting to individual points, e.g., membership inference.

Practically, this implies that FASD is especially useful in terms of privacy protection when sensitive features are not major joint contributors to prediction but can rather be seen as independent confounders or irrelevant to prediction altogether.

## Discussion

We show that FASD can provide SD with high utility in prediction tasks while maintaining privacy protection. This indicates its usefulness for publishing data across a wide range of scientific disciplines, which otherwise suffer from privacy concerns.

FASD achieves high utility by simplifying SD generation from complex heterogeneous datasets to simpler and more densely sampled sets of continuous representations. This encourages relevant features to be learned well and reduces the risk of overfitting to irrelevant patterns. However, due to its task-specific focus, FASD utility does not increase as much from increasing SD size as it does for other methods that are more suited to enhancing data diversity.

In terms of privacy protection, neglecting to learn irrelevant patterns promotes dissimilarity to RD and thereby potentially enhances privacy protection. However, FASD mitigates attribute inference risk mainly when sensitive features are either independent or non-contributors to extracted representations. Otherwise, it retains this risk through association with other (non-sensitive) features. Medical studies are an example where sensitive independent confounders are prevalent, e.g., age and gender, and FASD can be expected to adequately mitigate attribute inference. In other scenarios, FASD still provides the benefits of increased utility.

Next to introducing our method for SD generation, we hope that this article inspires researchers to think differently about SD. Currently, SD are considered to be of high quality when they have high fidelity to RD, while in many cases, this is the main culprit of privacy risk. Generating fit-for-purpose SD that are similar to RD *only* in ways contributing to their intended use may result in better privacy protection.

### Limitations of the study

We consider single prediction tasks, but for some datasets, more than one task may be of interest or even tasks of very different natures. In the case of multiple relevant prediction tasks, our method is extendable by computing representations in the context of multiple simultaneous predictions. However, whether generated SD are useful in this context depends entirely on whether input features have predictive power over all prediction objectives. For tasks different in nature, e.g., unsupervised tasks, we quickly move into territory where high fidelity is vital and FASD may not be the best solution.

We present FASD for tabular data, but it may be used for any type of data with proper adjustment to the architecture of the FASD encoder, e.g., implementing recurrent layers for sequential data or convolutional layers for spatial data. However, the method may not be sensible for certain data types where perceptual quality is typically required, like text or images. On the other hand, if only task-specific performance is required, FASD may still work well.

More generally, FASD is not a good solution in any scenario where high fidelity is the main objective. General examples of such scenarios are those where SD will be used for a wide variety of, momentarily, unknown tasks.

Furthermore, even when task-specific utility is all that matters, poor-fidelity SD from FASD may still hamper SD adoption. Low fidelity, which may be recognized by end-users, as they have some notion of what the RD looks like, may not inspire confidence that the SD are of sufficient quality to be used in their application.

Having mentioned these limitations, we still believe FASD has great potential for all scenarios where a specific set of prediction tasks is known to be of interest. In this case, FASD shows great promise to provide privacy-protective SD with high utility. Although we mainly focus on SD for sharing sensitive research data, this might also make FASD especially suitable to privacy-preserving federated learning by sharing SD across clients instead of model parameters.^4^ When aligning the prediction task in FASD to that of the federated learning algorithm, FASD has potential to generate and share useful data. Future research should investigate this by adapting the FASD training scheme to generate SD for subsets of RD (i.e., the clients) and benchmarking against other methods especially suited to privacy-preserving federated learning, e.g., dataset distillation.^51^^,^^52^

## Methods

### Challenges in SD generation

Generating SD to be simultaneously useful and protective of privacy turns out to be a difficult task. Typically, generative models minimize statistical divergence between SD and RD and thus produce SD that are statistically similar to RD. Examples vary from statistical methods like copulas^32^ to modern machine learning methods like generative adversarial nets (GANs)^53^ and VAEs.^16^ Although these methods produce SD that are statistically similar to RD and thus generally useful, this statistical similarity may come at a cost: an increased risk of reidentification through linkage or attribute inference.^9^

Many open challenges remain in the SD field, e.g., generating SD that are simultaneously realistic and diverse, propagating bias and quality issues from RD to SD, and a lack of standardized thresholds for SD quality and privacy metrics. In this article, we specifically focus on overcoming the utility-privacy trade-off in SD generation.^5^^,^^6^^,^^7^

The utility-privacy trade-off arises from the tension between generating SD that are useful and SD that are protective of privacy. Useful SD are typically similar to RD, i.e., they contain similar statistical properties. On the other hand, an increase in similarity increases reidentification risk.^9^ Interventions aimed at improving privacy, e.g., adding noise, removing features, or otherwise perturbing the dataset, typically reduce overall similarity and thus both general and task-specific usefulness.^5^ Here, the friction between usefulness and privacy becomes clear.

Most efforts generate SD for high fidelity and employ *post hoc* privacy metrics to show that, rather coincidentally, privacy is protected adequately as well. This approach is not sustainable, as in many cases reidentification risk from SD is actually considerable.^9^ More sustainable approaches incorporate mechanisms to ensure a good balance between SD usefulness and privacy within the SD generation process.

Differential privacy is a popular framework that allows user specification of privacy levels during the SD generation process. It quantifies the maximum contribution of individual data points to the output of an algorithm^35^ or, in our case, the influence of RD points on generated SD. Setting strong privacy constraints in differential privacy should thus ensure little reidentification risk, as it becomes hard to match SD to individual RD points. Generative models typically incorporate differential privacy by injecting noise during the training process, e.g., during gradient optimization in neural nets. Differential privacy has been implemented for a variety of models, e.g., GANs, VAEs, and Bayesian nets.^30^^,^^34^^,^^41^^,^^54^ Although it provides a user specification of privacy levels, injecting noise during model training typically comes at a cost in SD utility.^55^ Differential privacy is thus an apt way to *control* the utility-privacy trade-off, but it does not aim to *overcome* it.

Instead of differential privacy, Yoon et al.^22^ impose an “identifiability” constraint on the objective function of their GAN. This puts a constraint on the ratio between the distance to the closest SD and RD points. In other words, it constrains how similar individual SD points are allowed to be to individual RD points. The allowed similarity level is user specified, and typically lower levels correspond to better privacy protection, but also lower SD usefulness.^22^ These types of constraints on generative model objective functions are another way to control the utility-privacy trade-off in SD generation.

### FASD

Instead of simply controlling it, we wish to find a Pareto improvement on the utility-privacy trade-off, i.e., increase utility while retaining privacy protection. As a first step, we suggest a more nuanced view on SD utility. In many scenarios, SD are generated for data sharing with a specific task already in mind. In this case, fidelity metrics are irrelevant as long as utility in that task is high. Some articles even solely report utility metrics and omit fidelity metrics to indicate SD quality.^30^ Some general examples of such scenarios, where SD are generated with a specific task already in mind, are the following:

(1) SD of patients for developing medical devices, e.g., clinical decision-support systems^56^: the task is predicting disease risk based on health markers.
(2) SD of financial transactions for developing fraud detection models^57^: the task is predicting whether a transaction is fraudulent based on customer and transaction characteristics.
(3) SD of customers for developing personalized recommender systems^58^: the task is predicting which item the customer is likely to buy based on previous customer-item interactions.

Since utility metrics are the main concern here, we aim to improve privacy protection of SD without diminishing utility. Harming fidelity, however, is explicitly not a concern. Our methodology achieves this by compressing information from the RD to the underlying factors that contribute to the prediction task for which we optimize utility before generating SD. This way, SD propagate useful information from RD in the context of the task and ignore any other information, thereby decreasing similarity to RD and potentially decreasing reidentification risk. Information is compressed through representation learning in the context of a supervised prediction task, i.e., the task at hand for a given dataset.

#### Representation learning

Representation learning concerns itself with “learning representations of the data that make it easier to extract useful information when building classifiers or other predictors.”^15^ It was mainly popularized to extract useful information from complex high-dimensional data types where the original features are uninformative, e.g., text, image, and audio data, but is also increasingly being used for simpler structured data formats.^59^^,^^60^ Good representations are typically those that capture the posterior distribution of *latent factors*, rather than simply observed inputs, and are useful as input to a prediction model.^15^

Neural nets are a common choice in representation learning, since their architecture accommodates these two properties of good representations. First, hidden activations of neural nets consist of non-linear combinations of input features, which resemble the latent factors in this context. These activations are consequently used as input to the prediction head of the network, i.e., the prediction model. By sampling from the activations of the final hidden layer of a neural net, one can obtain data that resemble the posterior distribution of latent factors that are useful as input to a prediction model.

#### Generation process

Generating FASD is a two-step process. The first step transforms RD to representations in the context of a supervised prediction task, and the second step generates SD from these representations. Figure 1 provides a schematic overview.

To compute representations, we train a regular feedforward neural net to predict a target feature from input features. In this neural net we distinguish the encoder, i.e., all layers up until (but excluding) the prediction head, and the predictor, i.e., the prediction head. We compute representations by passing input features of RD through the encoder.

Representations from the previous step comprise the input to an SD generator in the second step to generate synthetic representations. A good choice for an algorithm here is any algorithm that can accurately model—and draw new samples from—the continuous distribution of the representations. We opt for a VAE, but diffusion models,^61^ for example, would be an equally valid choice. We recommend against GANs, which suffer from training stability issues like mode collapse more often,^62^ in which case the synthetic representations are not an accurate reflection of the true diversity of the underlying latent factors in the RD.

Last, we decode synthetic representations to the original feature space and thereby obtain SD. The decoder follows a similar (but flipped) architecture as the encoder and is trained to predict RD from the representations computed in the previous step. Note that the decoder aims to find the inverse transformation to the encoder, and when the encoder is exactly invertible (e.g., when using affine coupling layers), the inverted encoder may be used as decoder.

The encoder architecture determines the structure of the representations. A more complex encoder, e.g., using non-linear activations, might extract more information valuable to prediction but also complicates learning tasks for the VAE and decoder. For example, when using rectified linear units as activation, representations are zero inflated, which is not well suited to be learned by a VAE. Due to this trade-off, we achieved best results using linear activations (clamped to [−1,1] for stability)—although results using tanh were similar. We recommend selecting activation functions based on the complexity of your dataset, keeping the trade-off discussed above in mind.

Next to our proposed approach for FASD, we also experimented with a simpler strategy: encoding data to representations regularized by a Gaussian prior to be able to directly sample synthetic representations and decode these back to the original feature space, obviating the need for training a VAE. However, representations computed in this approach were a lot less stable due to the joint optimization task, and utility was not as high. Still, future research should look into further simplifying our approach for FASD.

### Benchmarking SD generators

We benchmark FASD against other SD generators, namely CTGAN, TVAE, AdsGAN, and DP-GAN. These methods are all based on GANs or VAEs.

#### GANs

GANs are generative deep learning methods consisting of a generator and discriminator neural net.^53^ The generator produces SD from randomly sampled noise, whereas the discriminator aims to distinguish whether the data it receives are RD or come from the generator. Crucially, the generator receives feedback only from the discriminator and is not trained on RD directly.

We train all GAN-based methods using Wasserstein loss to improve training stability.^63^

#### VAEs

VAEs are another class of generative deep learning methods consisting of an encoder and decoder neural net.^16^ The encoder maps RD to a latent space, after which the decoder aims to reconstruct the original input. Crucially, the latent space is regularized during training to mimic a standard normal distribution, such that SD can be generated by passing randomly sampled noise through the decoder.

#### CTGAN

Introduced in Xu et al.,^31^ CTGAN is a GAN-based SD generator that conditions on discrete features to handle their class imbalance and applies normalization per mode to deal with complex numerical features. The result is increased fidelity of generated SD.

#### TVAE

Also introduced in Xu et al.,^31^ TVAE is a VAE-based SD generator that applies conditioning and mode-specific normalization similar to CTGAN. It was introduced as a high-performing baseline to compare against CTGAN, since the SD generator in TVAE is built on RD directly, unlike in GAN-based methods like CTGAN, which usually results in higher fidelity to RD.

#### AdsGAN

Introduced in Yoon et al.,^22^ AdsGAN is a GAN-based SD generator that adds the identifiability metric (see Table 1) as an additional loss component during training. This way, SD is generated to have a user-specified identifiability level, which lets the user control the trade-off between SD fidelity and privacy.

#### DP-GAN

Introduced in Xie et al.,^34^ DP-GAN is a GAN-based SD generator that imposes differential privacy during training by adding noise (and clipping) per-sample gradients. Hereby, DP-GAN provides formal privacy guarantees, namely with parameter ϵ quantifying the influence of individual training samples on generated SD and parameter δ indicating the probability that the privacy guarantee of ϵ does not hold. Typically, values of 0<ϵ≤1 are thought to provide strong privacy guarantees.

We fix the standard deviation of noise to be added to the gradients σ=1 and clip the norm of per-sample gradients at 1. Dependent on the SD generator architecture and dataset, these lead to different levels of ϵ tracked by the privacy accountant,^64^ for which we report the average and standard deviation over the 10 training folds. Namely, ε=16.4±3.1 for adult, ϵ=22.5±4.6 for credit, ϵ=33.8±5.7 for student, and ϵ=45.9±13.0 for heart. We fix δ at $\frac{1}{n}$ for each dataset.

Interestingly, even for these relatively loose privacy budgets, the utility of SD from DP-GAN is quite low (see Table 4). Although good results have been achieved previously, e.g., on image data, results for complex mixed-type tabular datasets (as in this research) are often poor.^65^^,^^66^^,^^67^ Generating high-quality SD for complex mixed-type tabular datasets with strong differential privacy guarantees remains very much an open issue.

### Hyperparameter optimization

We employ Bayesian hyperparameter tuning to select suitable architectures for the neural nets contained within FASD and the benchmarking SD generators. Here, we use tree-based density estimators to learn the distribution of the hyperparameter space and subsequently sample a set of parameters that perform best in terms of TSTR performance, i.e., averaged over linear regression, XGBoost, and neural net performances. We tune the number of layers and nodes in each neural net and perform 32 trials for each SD generator. Additionally, the size of the VAE latent space is tuned for TVAE and FASD and the size of the representations in FASD.

Neural net layers are tuned between 1 and 3 and nodes and VAE latent space size between 50 and 250. The size of representations in FASD is tuned between 50 and 150. All SD generators are trained using early stopping, i.e., until there is no improvement on a validation set for 250 epochs. All other hyperparameters (e.g., learning rate, batch size, and dropout) are left as their default in the SynthCity library implementation. Only for small datasets, i.e., the heart dataset, we manually reduce the batch size of SD generators and the TSTR neural net to 32. For DP-GAN on the other hand, we increase batch sizes, as larger batches improve performance in differentially private training,^68^ to 64 for heart, 512 for student, and 1,024 for the credit and adult dataset.

Table 6 shows the final neural net architectures found through Bayesian tuning.

**Table 6:** Architectures of synthetic data generators after Bayesian search

SD generator	Component	Parameter	Adult	Credit	Student	Heart
AdsGAN	generator	layers	2	3	1	2
		nodes	250	100	250	50
	discriminator	layers	2	3	2	2
		nodes	250	100	50	50
CTGAN	generator	layers	2	3	1	3
		nodes	150	50	150	50
	discriminator	layers	3	3	3	1
		nodes	150	50	100	200
FASD	encoder (VAE)	layers	2	2	1	3
		nodes	200	200	250	100
	decoder (VAE)	layers	3	3	1	3
		nodes	150	50	150	150
	embedding (VAE)	units	250	200	50	50
	representation	units	150	50	150	50
DP-GAN	generator	layers	3	2	2	3
		nodes	150	250	50	100
	discriminator	layers	2	1	1	2
		nodes	200	150	100	200
TVAE	encoder	layers	1	3	2	2
		nodes	50	100	250	100
	decoder	layers	3	2	3	3
		nodes	200	100	200	100
	embedding	units	50	50	50	100

### SD evaluation

We employ a wide variety of evaluation metrics to assess SD quality from FASD versus other SD generators. Table 1 provides short summaries on the metrics; in this section we provide additional explanation.

#### Fidelity

##### JS distance

JS distance is a bounded (i.e., on [0,1]) and symmetrized version of Kullback-Leibler divergence and measures the similarity between two probability distributions. Low JS distance thus indicates statistical similarity between SD and RD.

##### α-precision and β-recall

Although JS provides a measure for distributional similarity, it lumps different failure modes of SD into a single metric. Precision-recall analyses for generative models indicate the degree to which the SD distribution *is covered by* the RD distribution (precision) and the degree to which SD distribution *covers* the RD distribution (recall).^69^ Both are measures between 0 and 1, where 1 indicates full coverage and 0 indicates no coverage. Precision recall may help to indicate issues such as overfitting, when SD are realistic but not diverse (high precision, low recall), and underfitting, when SD are diverse but not realistic (low precision, high recall).

With α-precision and β-recall, Alaa et al.^7^ go a step further and assess whether SD fall within α-support of the RD distribution and whether RD falls within β-support of the SD distribution. Here, α- and β-support indicate the minimum volume subset, which contains a probability mass of α and β, respectively. Intuitively, these metrics indicate whether a sample is both *realistic* and *typical*, meaning it falls within the support *and* within a dense region of the RD distribution.

##### Distinguishability

If SD and RD can be accurately distinguished from each other, this indicates they are not similar. This problem can be formulated as a binary classification task where SD and RD receive opposite binary labels, and a classifier is trained to predict them.

We use an XGBoost^70^ classifier and report the area under the receiver operating characteristic curve (AUROC) on an independent test set. Here, an AUROC close to 1 indicates perfect distinguishability, whereas an AUROC close to 0.5 indicates no distinguishability.

#### Utility

##### TSTR

Esteban et al.^33^ formally introduce an approach to compare the performances of predictive models trained on SD and RD on a test set of RD, as TSTR. As it is generally unknown which prediction model will be employed by the end-user of SD, we assess the TSTR approach for a variety of prediction models, namely linear regression, XGBoost, and a feedforward neural net.

##### Feature importance rank distance

In many prediction tasks, not only the predictions themselves but also how input features lead to the predictions are relevant. In this case, high-utility SD and RD should provide similar answers to this question.

To measure this, we provide the correlation, i.e., Kendall’s τ, between feature importances extracted from an XGBoost model fit on SD and RD. High correlation indicates that the importance ranks of input features are similar between SD and RD, and features are thus (relatively) equally important.

#### Privacy

##### *k-*map

In k-map, for each individual in a dataset, at least k individuals from a reference dataset are similar based on sensitive features.^20^ In this scenario, the dataset is RD and the reference dataset is SD. Higher values of k indicate less risk of matching SD points to individual RD points.

##### δ-presence

In δ-presence, for each individual in a sensitive dataset, the ratio of similar individuals (based on sensitive features) in this same dataset to similar individuals from a reference dataset is at most δ.^21^ High values of δ indicate a *relatively* low amount of similar individuals from the reference dataset to each individual in a sensitive dataset. In this scenario, RD are the sensitive dataset and SD are the known reference dataset.

##### Authenticity

Although originally introduced as a type of fidelity metric,^7^ the authenticity score can also be interpreted as a privacy metric. The metric computes the frequency at which an RD point is closer, e.g., by Euclidean distance, to an RD point than any SD point. A higher score indicates less one-to-one similarity between RD and SD. In terms of fidelity, this indicates good “generalizability,” meaning that SD is not overfitting to individual RD points. In terms of privacy, this indicates less one-to-one matching and thus reidentification risk.

##### Identifiability

The identifiability score^22^ computes the frequency at which an SD point is closer to an RD point than any other RD point. Note that this is opposite to the authenticity score. Also, instead of simply calculating Euclidean distance, distances are calculated by first weighting each column by the entropy of its unique values, ensuring rare values are seen as more identifiable.

##### Attribute inference

Next to similarity of individual SD to RD points, another privacy risk is malicious parties launching inference attacks using SD. For example, they can train a predictive model to infer sensitive features from non-sensitive features, for which RD may be available to them. Then, using the model trained on SD, they may infer sensitive features in RD. We launch such attribute inference attacks on generated SD ourselves using XGBoost models and assess their accuracy. As predictive accuracy metrics, we use AUROC for discrete features and $R^{2}$ score for numerical features. High accuracy in attribute inference attacks indicates that those attributes may be accurately inferred.

##### Membership inference

To determine which RD points may have been used to train SD generators, malicious parties may launch membership inference attacks. Here, we use DOMIAS^23^ as inference attack setup. Simply put, it estimates the relative density of SD versus some reference RD, where density peaks correspond to “unexpectedly” high SD density. In turn, this indicates (local) overfitting to RD points. Originally, van Breugel et al.^23^ used block neural autoregressive flows^71^ for density estimation in DOMIAS. However, both from our own experiments and from the literature, this has been noted to work poorly for complex mixed-type datasets.^66^^,^^72^^,^^73^ Future work should address adapting these autoregressive flows to mixed-type base distributions to better accommodate these types of datasets.

As an alternative, we employ VAEs for density estimation, or more specifically, we use their loss as a proxy for likelihood as it is known to provide a lower bound to the data log likelihood.^16^ Note that using VAEs for detecting overfitting might favorably bias the VAE-based benchmarks, e.g., TVAE. We use two hidden layers of 250 nodes in both the encoder and the decoder and a latent size of 128.

Furthermore, in our experiments, the reference RD correspond to half of the RD test set, and membership predictions are made for the other half of the RD test set (non-members) and the RD training set (members), for which we report the AUROC.

## Resource availability

### Lead contact

All questions can be directed to the lead contact, Jim Achterberg (j.l.achterberg@lumc.nl).

### Materials availability

The data generated in this study can be replicated through the openly available source code and data.

### Data and code availability

Our source code is archived at Zenodo^74^: https://doi.org/10.5281/zenodo.15000502. The data are openly available at the UCI ML repository^37^: https://archive.ics.uci.edu/datasets.

## Acknowledgments

This work is co-funded by the HORIZON.2.1 – Health Programme of the 10.13039/501100000780European Commission, grant agreement no. 101095661 – Innovative applications of assessment and assurance of data and synthetic data for regulatory decision support (INSAFEDARE).

## Author contributions

J.A. and M.H. contributed to the conception and design of the study. J.A. conducted the technical analyses. J.A. led the writing of the manuscript. M.H. and B.v.D. wrote the manuscript. M.S. revised the manuscript and helped to shape the research, analyses, and manuscript. All authors read and approved the final manuscript.

## Declaration of interests

The authors declare no competing interests.
